# Author Correction: An evaluation of the accuracy and speed of metagenome analysis tools

**DOI:** 10.1038/s41598-020-63176-4

**Published:** 2020-04-20

**Authors:** Stinus Lindgreen, Karen L. Adair, Paul P. Gardner

**Affiliations:** 1 Biomolecular Interaction Centre, University of Canterbury, Christchurch, New Zealand; 2 School of Biological Sciences, University of Canterbury, Christchurch, New Zealand; 3 Department of Biology, Section for Computational and RNA Biology, University of Copenhagen, Copenhagen, Denmark; 4 Present Address: Carlsberg Laboratory, Gamle Carlsberg Vej 4-10, 1799 Copenhagen V, Denmark

Correction to: *Scientific Reports* 10.1038/srep19233, published online 18 January 2016

The website on which the data underlying this study was stored is no longer maintained. The data is now deposited in zenodo, with a 10.5281/zenodo.3662895. As such, in the Abstract,

“Data sets and results are freely available from http://www.ucbioinformatics.org/metabenchmark.html”

should read:

“Data sets and results are freely available from https://zenodo.org/record/3662895#.XkMnZnUzbRI”

