# Supplementary material for: An evaluation of the accuracy and speed of metagenome analysis tools
Source: Sci Rep. 2016 Jan 18;6:19233. doi: 10.1038/srep19233 (PMC4726098; doi:10.1038/srep19233)
Supplement: Supplementary Information [file srep19233-s1.pdf]

Supplementary material for "An evaluation of the accuracy and speed of metagenome analysis tools"

Stinus Lindgreen[1,2,3,$*], Karen L. Adair[1,2], Paul P. Gardner[1,2]
[1]Biomolecular Interaction Centre, University of Canterbury, Christchurch, New Zealand
[2]School of Biological Sciences, University of Canterbury, Christchurch, New Zealand
[3]Section for Computational and RNA Biology, Department of Biology, University of Copenhagen, Copenhagen, Denmark
[*]Corresponding author: stinus@binf.ku.dk
[$]Current address: Carlsberg Laboratory, Gamle Carlsberg Vej 4-10, 1799 Copenhagen V, Denmark

Section 1: Supplementary table and figure legends:

Table S1: The real and predicted proportions of phyla per method.

Table S2: The genomes used for simulating the metagenomes. Column 1: Phylum, column 2: EMBL ID, column 3: Details on the exact strain/species used.

Table S3: The predicted and expected functional shifts.

Figure S1: Barplot of the relative abundance of phyla predicted by each method. Methods are color coded.

Figure S2: The $\log_2$ odds score between the predicted and actual relative abundance for each phylum and each tool, i.e. $\log_2$(predicted_abundance/known_abundance). A positive log odds score means an overrepresentation, and a negative log odds score means an underrepresentation. A difference of 1 in log odds score means a two-fold difference.

Figure S3: Sum of log odds scores for all methods including the Eukaryote component for the methods that predicted (some) Eukaryotes.

Figure S4: NMDS plot showing the similarities between each metagenome based on the predicted relative abundances. All phyla (including Eukaryotes but excluding "Other" i.e. phyla not included in the test set) are used in this plot.

Section 2: Command lines

Below are listed the command lines and additional steps if applicable used for each method. For clarity, paths etc. have been removed from the commands, and generic filenames are used (e.g. FILE1.)

Section 2.1: Genometa 0.51

Input: Paired end Fasta files, FILE1.FASTA and FILE2.FASTA

Step 1: Use bowtie 1.1.0 to map reads to Genometa database “allgenomes_april_2012_v6_one_per_genus. Use settings recommended by the manual. Utilize multiple cores:

```
bowtie -t allgenomes_april_2012_v6_one_per_genus --sam -p 8 --maxins
500 -n 3 -l 40 -e 200 --best -f -1 FILE1.FASTA -2 FILE2.FASTA
OUTPUT.SAM
```

Step 2: Open Genometa. Convert SAM file to BAM.

Step 3: Export CSV file with mapping information.

Section 2.2: Kraken 0.10.4 beta

Input: Gzipped, paired-end fastq files, FILE1.FASTQ.GZ and FILE2.FASTQ.GZ

Step 1: Map reads to the Kraken database:

```
kraken --db kraken-db --fastq-input --gzip-compressed --output
OUTPUT.kraken.out --paired FILE1.FASTQ.GZ FILE2.FASTQ.GZ
```

Step 2: Generate Kraken report:

```
kraken-report --db kraken-db OUTPUT.kraken.out > OUTPUT.kraken.report
```

Step 3: Generate mpa report:

```
kraken-mpa-report --db kraken-db OUTPUT.kraken.out >
OUTPUT.kraken.mpa-report
```

Section 2.3: LMAT 1.2.4

Input: Fasta file with concatenated read pairs, INPUT.FASTA

Step 1: Put run time input in your path:

```
export LMAT_DIR=PATH/runtime_inputs
```

Step 2: Assign taxonomic labels to each read:

```
run_rl.sh --db_file=PATH/kML.v4-14.20.g10.db
--query_file=INPUT.FASTA --odir=PATH/output_dir --threads=8
```

Step 3: Assign gene identifiers to reads:

```
find output_dir/ -name
INPUT.FASTA.kML.v4-14.20.g10.db.lo.rl_output[0-9]\*.out >
output_dir/INPUT.rl_output.flst
```

```
run_gl.sh --db_file=PATH/lmat.genes.7-14.db
--ilst=output_dir/INPUT.rl_output.flst --odir=output_dir
```

Step 4: Content summary:

```
run_cs.sh --ilst=output_dir/INPUT.rl_output.flst --odir=output_dir
--filesum=output_dir/rl_output.flst.lmat.genes.7-14.db.gl_output.0.1.
20.genesummary

run_cs.sh --ilst=output_dir/INPUT.rl_output.flst --odir=output_dir
--filesum=output_dir/INPUT.FASTA.kML.v4-14.20.g10.db.lo.rl_output.0.3
0.fastsummary
```

Step 5: Analyze functional content of data sets using scripts and mapping files obtained from the authors:

```
./LMAT2multi-genesummaryTable.pl -in FILE.list -out
FILE.LMAT.gene_annot.SEED.megan
```

Where FILE.list contains one line:
ID<TAB><PATH TO .genesummary FILE>

Step 6: Analyze output using MEGAN.

Functional analysis: The following SEED subsystems were used:

**Photosynthesis**	**Nitrogen fixation**	**Pathogens**
Photosynthesis; Electron transport and photophosphorylation	Nitrogen Metabolism; Nitrogen fixation	Virulence, Disease and Defense; Invasion and intracellular resistance
Photosynthesis; Light-harvesting complexes		Virulence, Disease and Defense; Toxins and superantigens
		Virulence, Disease and Defense; Adhesion
		Virulence, Disease and Defense; Streptococcus pyogenes Virulome
		Motility and Chemotaxis; Flagellar motility in Prokaryota
		Motility and Chemotaxis; Bacterial Chemotaxis

		Virulence; Type III, Type IV, Type VI, ESAT secretion systems
		Regulation and Cell signaling; Quorum sensing and biofilm formation
		Dormancy and Sporulation; Spore germination
		Phages, Prophages, Transposable elements, Plasmids; Pathogenicity islands

Section 2.4: MetaPhlAn 2.0

Input: Gzipped, paired-end FastQ files, FILE1.FQ.GZ and FILE2.FQ.GZ

Step 1: Run the MetaPhlAn Python script:

```
python2.7 metaphlan2.py FILE1.FQ.GZ,FILE2.FQ.GZ --mpa_pkl
mpa_v20_m200.pkl --bowtie2db mpa_v20_m200 --input_type fastq
--bowtie2out FILE.bowtie2.bz2 --nproc 8 --output_file
FILE.metaphlan2.out
```

Section 2.5: MEGAN 5.7.0

Input: Fasta files mapped to RefSeq release 66 using Diamond. SAM files analyzed with MEGAN.

Step 1: Map the two Fasta files to RefSeq using Diamond. The mapper does not use paired information:

```
diamond blastx --db refseq_66 --query FILE1.fasta --sam FILE1.sam"
--threads 20
diamond blastx --db refseq_66 --query FILE2.fasta --sam FILE2.sam"
--threads 20
```

Step 2: Analyze SAM files using MEGAN. Files with MEGAN commands were used to run from the command line, and a file mapping RefSeq to tax ID was provided by the MEGAN authors:

```
load taxGIFile='/data/bin/megan/gi_taxid_prot.bin';
load keggGIFile='/data/bin/megan/gi_taxid_prot.bin';
load seedGIFile='/data/bin/megan/gi_taxid_prot.bin';
import blastFile='FILE1.sam', 'FILE2.sam' meganFile='FILE.rma'
maxMatches=200 minScore=30.0 maxExpected=0.001 topPercent=10.0
minSupport=5 minComplexity=-1.0 useMinimalCoverageHeuristic=false
```

```
useSeed=true useCOG=false useKegg=true paired=true suffix1='/1'
suffix2='/2' useIdentityFilter=false textStoragePolicy=Embed
blastFormat=SAM
mapping='Taxonomy:BUILT_IN=true,Taxonomy:GI_MAP=true,SEED:GI_MAP=true
,KEGG:GI_MAP=true';
```

Functional analysis: The following SEED subsystems were used.

Photosynthesis	Nitrogen fixation	Pathogens
Photosynthesis; Light-harvesting complexes	None	Virulence, Disease and Defense; Adhesion
		Virulence, Disease and Defense; Streptococcus pyogenes Virulome
		Motility and Chemotaxis; Flagellar motility in Prokaryota
		Motility and Chemotaxis; Bacterial Chemotaxis
		Virulence; Type III, Type IV, Type VI, ESAT secretion systems
		Regulation and Cell signaling; Quorum sensing and biofilm formation
		Dormancy and Sporulation; Spore germination
		Phages, Prophages, Transposable elements, Plasmids; Pathogenicity islands

Section 2.6: MG-RAST 3.3.6

Input: Gzipped, paired-end FastQ files uploaded to the webserver. A complete metadata file was provided. Read pairs were merged if possible, with non-merged reads retained for analysis. Pipeline options were kept as default.

For analysis, the API was used as instructed by the authors:

Step 1: Extract taxonomic information at the phylum level

```
python mg-abundant-taxa.py --id <MG-RAST ID> --token <WEBKEY> --level
phylum --source M5NR --top 100000 --evalue 1 > ID.phylum.tsv
```

Step 2: Extracting functional classification from SEED

```
python mg-abundant-functions.py --id <MG-RAST ID> --token <WEBKEY> --level <LEVEL> --source Subsystems --top 100000 --evalue 1 > ID.LEVEL.SEED
```

Step 3: Extract the number of shuffled reads that mapped to something

```
curl "http://api.metagenomics.anl.gov/annotation/similarity/<MG-RAST ID>?identity=80&type=organism&source=RefSeq&auth=<WEBKEY>" | grep -i Random > ID.shuffled.reads
```

Functional analysis: The following SEED subsystems were used:

**Photosynthesis**	**Nitrogen fixation**	**Pathogens**
Photosynthesis; Electron transport and photophosphorylation	Nitrogen Metabolism; Nitrogen fixation	Dormancy and Sporulation; Spore DNA protection
Photosynthesis; Light-harvesting complexes		Dormancy and Sporulation; Spore germination
		Motility and Chemotaxis; Bacterial Chemotaxis
		Motility and Chemotaxis; Flagellar motility in Prokaryota
		Phages, Prophages, Transposable elements, Plasmids; Pathogenicity islands
		Regulation and Cell signaling; Quorum sensing and biofilm formation
		Virulence, Disease and Defense; Adhesion
		Virulence, Disease and Defense; Invasion and intracellular resistance
		Virulence, Disease and Defense; Streptococcus pyogenes Virulome
		Virulence, Disease and Defense; Toxins and superantigens

Section 2.7: QIIME 1.8.0 / PICRUSt 1.0.0

Input: QIIME was not developed for the analysis of shotgun metagenomes and is not optimized for handling the fragmented rRNA reads you would expect from shotgun sequencing. It is included in this benchmark because it is a widely used tool for the analysis of targeted rRNA sequences, and we wanted to evaluate if it can be used outside its area. The input is generated by scanning Fasta files for each our metagenomes for 16S rRNA sequences and using those as input. Three sets of output were generated (using read 1 alone; using read 2 alone; and using both reads) and analyzed, but the result did not differ.

Step 1: Scan for bacterial 16S SSU rRNA (Rfam model RF00177) and archaeal 16S SSU rRNA (Rfam model RF01959) using the equivalent hmm:

```
nhmmer --noali -E 0.001 --incE 0.001 --cpu 20 RF00177 FILE.fasta > FILE.bacteria.out
nhmmer --noali -E 0.001 --incE 0.001 --cpu 20 RF01959 FILE.fasta > FILE.archaea.out
```

Step 2: Use custom scripts to extract the corresponding reads and turn them into Fasta files, rRNA.FASTA.

Step 3: Use QIIME to pick OTUS in GreenGenes ver. 13.8 at the 97% identity level:

```
pick_otus.py -i FILE.FASTA -s 0.97 -m uclust_ref -r greengenes/gg_13_8_otus/rep_set/97_otus.fasta
```

Step 4: Pick representative sequences:

```
pick_rep_set.py -i uclust_ref_picked_otus/FILE_otus.txt -f FILE.fasta -o FILE.REP.FASTA
```

Step 5: Assign taxonomy:

```
assign_taxonomy.py --uclust_similarity=0.97 -i FILE.REP.FASTA -r greengenes/gg_13_8_otus/rep_set/97_otus.fasta -t greengenes/gg_13_8_otus/taxonomy/97_otu_taxonomy.txt
```

Step 6: Make OTU table:

```
make_otu_table.py -i uclust_ref_picked_otus/FILE_otus.txt -t uclust_assigned_taxonomy/FILE.REP_set_tax_assignments.txt -o FILE.otu_table.biom
```

Step 7: Summarize data:

```
summarize_taxa.py -a -L 1,2,3,4,5,6,7 -i FILE.otu_table.biom -o taxonomy_summaries
```

Step 8: For functional inference, PICRUSt ver. 1.0.0 was used as follows. First, the BIOM table from step 6 was filtered to contain only reference OTUs:

```
filter_otus_from_otu_table.py -i FILE.otu_table.biom -o
FILE.closed_ref_otu_table.biom --negate_ids_to_exclude -e
greengenes/gg_13_8_otus/rep_set/97_otus.fasta
```

Step 9: The filtered BIOM table was normalized:

```
normalize_by_copy_number.py -i FILE.closed_ref_otu_table.biom -o
FILE.normalized_otus.biom
```

Step 10: Predict functional profiles of metagenomes:

```
predict_metagenomes.py -i FILE.normalized_otus.biom -o
FILE.metagenome_predictions.biom
```

Step 11: Function was summarized for KEGG pathways at level 3:

```
categorize_by_function.py -f -i FILE.metagenome_predictions.biom -c
KEGG_Pathways -l 3 -o FILE.metagenome_predictions.KEGG.L3.txt
```

For functional analysis, the following KEGG pathways are used:

**Photosynthesis**	**Nitrogen fixation**	**Pathogens**
Metabolism; Energy Metabolism; Photosynthesis	Metabolism; Energy Metabolism; Nitrogen metabolism	Human Diseases; Infectious Diseases; Vibrio cholerae pathogenic cycle
Metabolism; Energy Metabolism; Photosynthesis - antenna proteins		Human Diseases; Infectious Diseases; Vibrio cholerae infection
Metabolism; Energy Metabolism; Photosynthesis proteins		Human Diseases; Infectious Diseases; Tuberculosis
		Human Diseases; Infectious Diseases; Staphylococcus aureus infection
		Unclassified; Cellular Processes and Signaling; Sporulation
		Human Diseases; Infectious Diseases; Shigellosis
		Human Diseases; Infectious Diseases; Pertussis
		Human Diseases; Infectious

		Diseases; Pathogenic Escherichia coli infection
		Unclassified; Cellular Processes and Signaling; Germination
		Cellular Processes; Cell Motility; Flagellar assembly
		Cellular Processes; Cell Motility; Bacterial motility proteins
		Cellular Processes; Cell Motility; Bacterial chemotaxis
		Environmental Information Processing; Membrane Transport; Bacterial secretion system
		Environmental Information Processing; Signaling Molecules and Interaction; Bacterial toxins

Section 2.8: mOTU

Input: Gzipped, paired-end Fastq files, FILE1.FASTQ.GZ and FILE2.FASTQ.GZ

Step 1: Run mOTU:

```
perl mOTUs.pl FILE1.FASTQ.GZ FILE2.FASTQ.GZ --processors=16
--output-directory=OUT
```

Step 2: For phylum abundances, the following output file was used:

```
0.taxonomic.profile.reads.extracted.mOTU.v1.padded.solexaqa.on.RefMG.
v1.padded.solexaqa.allbest.l45.p97.insert.mm.dist.among.unique.scaled
.phylum
```

Section 2.9: MetaPhyler 1.25

Input: Paired-end Fasta files, FILE1.FASTA and FILE2.FASTA analyzed separately but combined in the end.

Step 1: Analyze both files using BLASTN:

```
runMetaphyler.pl FILE1.FASTA blastn FILE1.blastn 16
runMetaphyler.pl FILE2.FASTA blastn FILE1.blastn 16
```

Step 2: Analyze both files using BLASTX:

```
runMetaphyler.pl FILE1.FASTA blastx FILE1.blastx 16
runMetaphyler.pl FILE2.FASTA blastx FILE1.blastx 16
```

Step 3: Combine all four output files:

```
combine FILE1.blastn.classification FILE1.blastx.classification
FILE2.blastn.classification FILE2.blastx.classification >
FILE.combined.classification
```

Step 4: Generate taxonomic profiles:

```
taxprof 0.9 FILE.combined.classification FILE.combined.taxprof
```

Section 2.10: Taxator-tk 1.2.1

Input: Unzipped, paired-end Fasta files, FILE1.FASTA and FILE2.FASTA, treated separately but combined in the end as instructed by the authors. Used the mapper “LAST” and the latest microbial reference database “nonredundant-microbial_20140513”.

Step 1: Analyze both files using Taxator-tk:

```
export
TAXATORTK_TAXONOMY_NCBI=./refdata/nonredundant-microbial_20140513/ncb
i-taxonomy
binning-workflow-fasta-last.bash FILE1.FASTA
binning-workflow-fasta-last.bash FILE2.FASTA
```

Step 2: As instructed by the authors, the two gff3-files are combined to use both sets of reads. First, remove identifier for pairs in gff3-files:

```
echo "##gff-version 3" > FILE.taxator_combined.gff3
grep -v "^##" FILE1.FASTA.0/sample.gff3 | sed ‘s/\/1//’ > FILE.tmp1
grep -v "^##" FILE2.FASTA.0/sample.gff3 | sed ‘s/\/2//’ > FILE.tmp2
sort -mk1,1 FILE.tmp1 FILE.tmp2 >> FILE.taxator_combined.gff3
```

Step 3: Reanalyze new combined gff3 files:

```
binner -f FILE.taxator_combined.gff3 -l FILE.taxator_combined.log >
FILE.taxator_combined.tax
taxknife -f2 --mode annotation -s path < FILE.taxator_combined.tax >
FILE.taxator_combined.tax-path.txt
grep ";" FILE.taxator_combined.tax-path.txt | cut -f2 | sed -e
's/[A-Za-z]\+;//' -e 's/;.*//' | sort | uniq -c >
FILE.taxator_combined.phylum.summary
```

Section 2.11: EBI Metagenomics

Input: Paired end fastq files were uploaded to the EBI servers and analyzed using the default pipeline.

Output: The .biom files were used for analyzing taxonomy, using QIIME to summarize taxa at the phylum level. Functional analysis was done using the GO.csv files and the following GO terms:

**Photosynthesis**	**Nitrogen fixation**	**Pathogens**
GO:0015979 photosynthesis biological process	GO:0009399 nitrogen fixation biological process	GO:0009405 pathogenesis biological process
		GO:0009403 toxin biosynthetic process biological process
		GO:0001539 ciliary or flagellar motility biological process
		GO:0009372 quorum sensing biological process
		GO:0030435 sporulation resulting in formation of a cellular spore biological process

Section 2.12: CLARK v1.1.3

Input: Paired-end Fastq files, FILE1.FASTQ and FILE2.FASTQ. Ran the sensitive version of the tool as instructed by the authors against the bacterial database.

Step 1: Set up database for phylum level analysis:

```
./set_targets.sh ./db bacteria --phylum
```

Step 2: Analyze files using CLARK:

for id in setA1 setA2 setA3 setB1 setB2 setB3; do echo $id; time

```
./classify_metagenome.sh -P FILE1.FASTQ  FILE2.FASTQ -R FILE.CLARK -n 60 -m 0 -k 20
```

Step 3: Filter results to get high confidence phylum level predictions:

```
./estimate_abundance.sh -D ./db -F FILE.CLARK.csv --highconfidence > FILE.phylum.csv
```

Section 2.13: One Codex

The zipped fastq files were uploaded and analyzed by the team behind the webserver. The files containing read level information on mapping were downloaded and analyzed by counting the number of reads mapped to each phylum.

Section 2.14: GOTTCHA 1.0a

Input: Paired-end fastq files, FILE1.FASTQ and FILE2.FASTQ.

Step 1: Analyze fastq files and directly obtain taxonomic distribution in FILE.tsv:

```
./bin/gottcha.pl --threads 60 --outdir ./ --input
FILE1.FASTQ,FILE2.FASTQ --database
./database/GOTTCHA_BACTERIA_c3514_k24_u24.species
```

Section 3: Notes on results

- Tenericutes are combined with Firmicutes to make all methods comparable.
- Whenever one or more phyla are excluded in a comparison, the relative abundances are normalized to make them comparable.

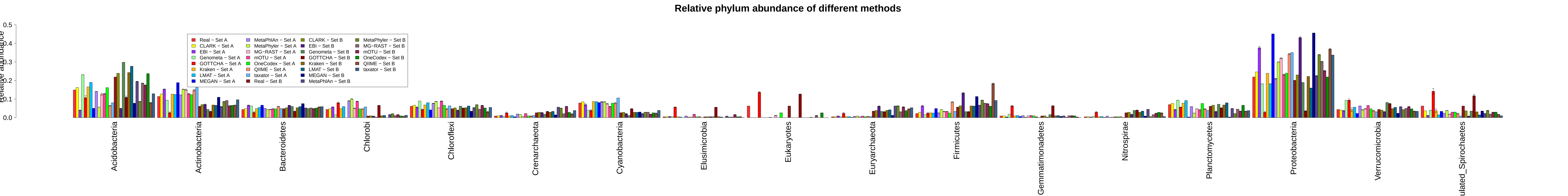

Relative phylum abundance of different methods
Relative abundance
Real − Set A
CLARK − Set A
EBI − Set A
Genometa − Set A
GOTTCHA − Set A
Kraken − Set A
LMAT − Set A
MEGAN − Set A
MetaPhlAn − Set A
MetaPhyler − Set A
MG−RAST − Set A
mOTU − Set A
OneCodex − Set A
QIIME − Set A
taxator − Set A
Real − Set B
CLARK − Set B
EBI − Set B
Genometa − Set B
GOTTCHA − Set B
Kraken − Set B
LMAT − Set B
MEGAN − Set B
MetaPhlAn − Set B
MetaPhyler − Set B
MG−RAST − Set B
mOTU − Set B
OneCodex − Set B
QIIME − Set B
taxator − Set B
Acidobacteria
Actinobacteria
Bacteroidetes
Chlorobi
Chloroflexi
Crenarchaeota
Cyanobacteria
Elusimicrobia
Eukaryotes
Euryarchaeota
Firmicutes
Gemmatimonadetes
Nitrospirae
Planctomycetes
Proteobacteria
Verrucomicrobia
ulated_Spirochaetes

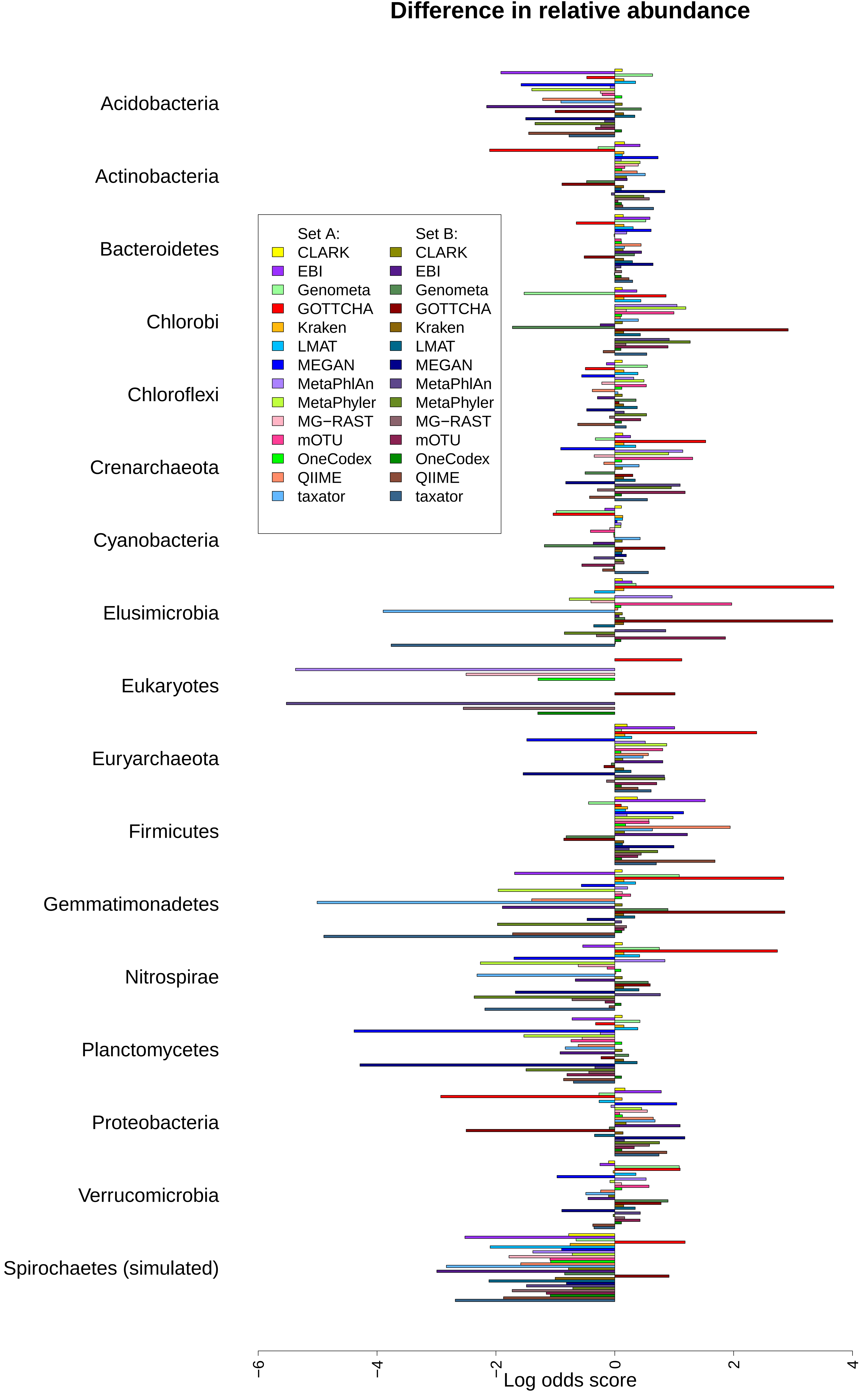

Difference in relative abundance
Acidobacteria
Actinobacteria
Bacteroidetes
Chlorobi
Chloroflexi
Crenarchaeota
Cyanobacteria
Elusimicrobia
Eukaryotes
Euryarchaeota
Firmicutes
Gemmatimonadetes
Nitrospirae
Planctomycetes
Proteobacteria
Verrucomicrobia
Spirochaetes (simulated)
Set A:
CLARK
EBI
Genometa
GOTTCHA
Kraken
LMAT
MEGAN
MetaPhlAn
MetaPhyler
MG−RAST
mOTU
OneCodex
QIIME
taxator
Set B:
CLARK
EBI
Genometa
GOTTCHA
Kraken
LMAT
MEGAN
MetaPhlAn
MetaPhyler
MG−RAST
mOTU
OneCodex
QIIME
taxator
−6
−4
−2
0
2
4
Log odds score

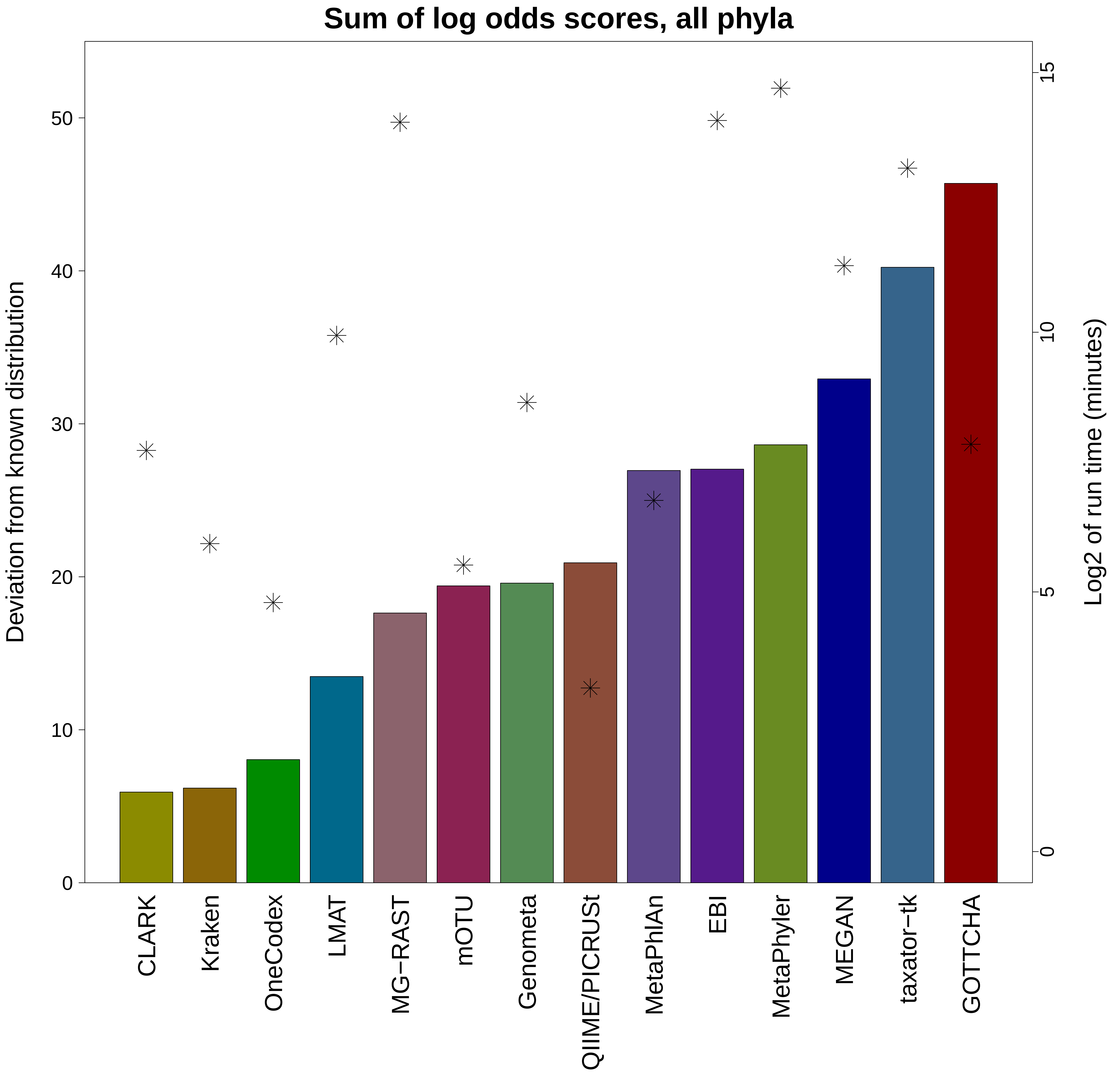

Sum of log odds scores, all phyla
Deviation from known distribution
Log2 of run time (minutes)
0
10
20
30
40
50
0
5
10
15
CLARK
Kraken
OneCodex
LMAT
MG−RAST
mOTU
Genometa
QIIME/PICRUSt
MetaPhlAn
EBI
MetaPhyler
MEGAN
taxator−tk
GOTTCHA

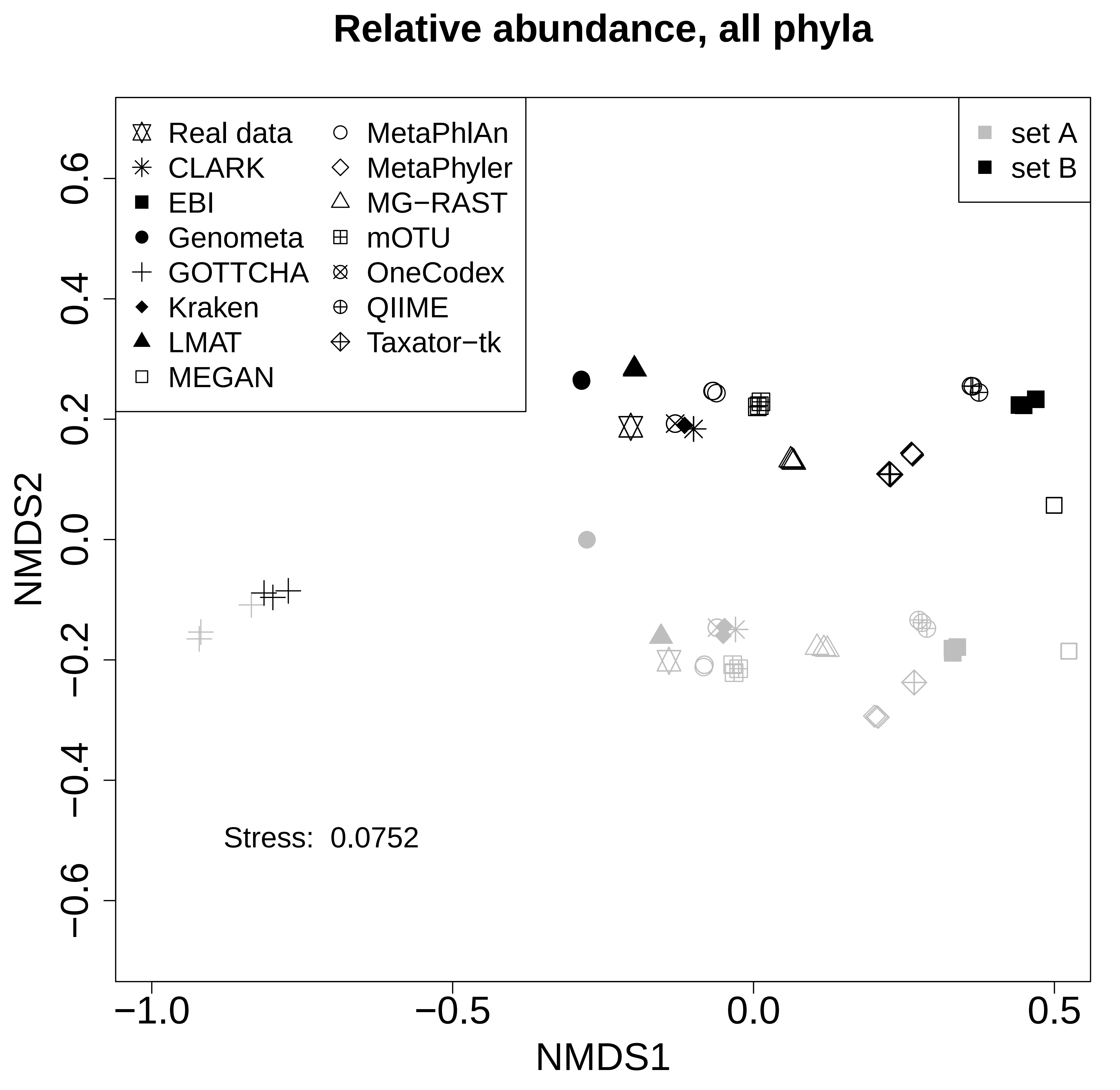

Relative abundance, all phyla
Real data
CLARK
EBI
Genometa
GOTTCHA
Kraken
LMAT
MEGAN
MetaPhlAn
MetaPhyler
MG−RAST
mOTU
OneCodex
QIIME
Taxator−tk
set A
set B
Stress: 0.0752
NMDS1
NMDS2
−1.0
−0.5
0.0
0.5
−0.6
−0.4
−0.2
0.0
0.2
0.4
0.6

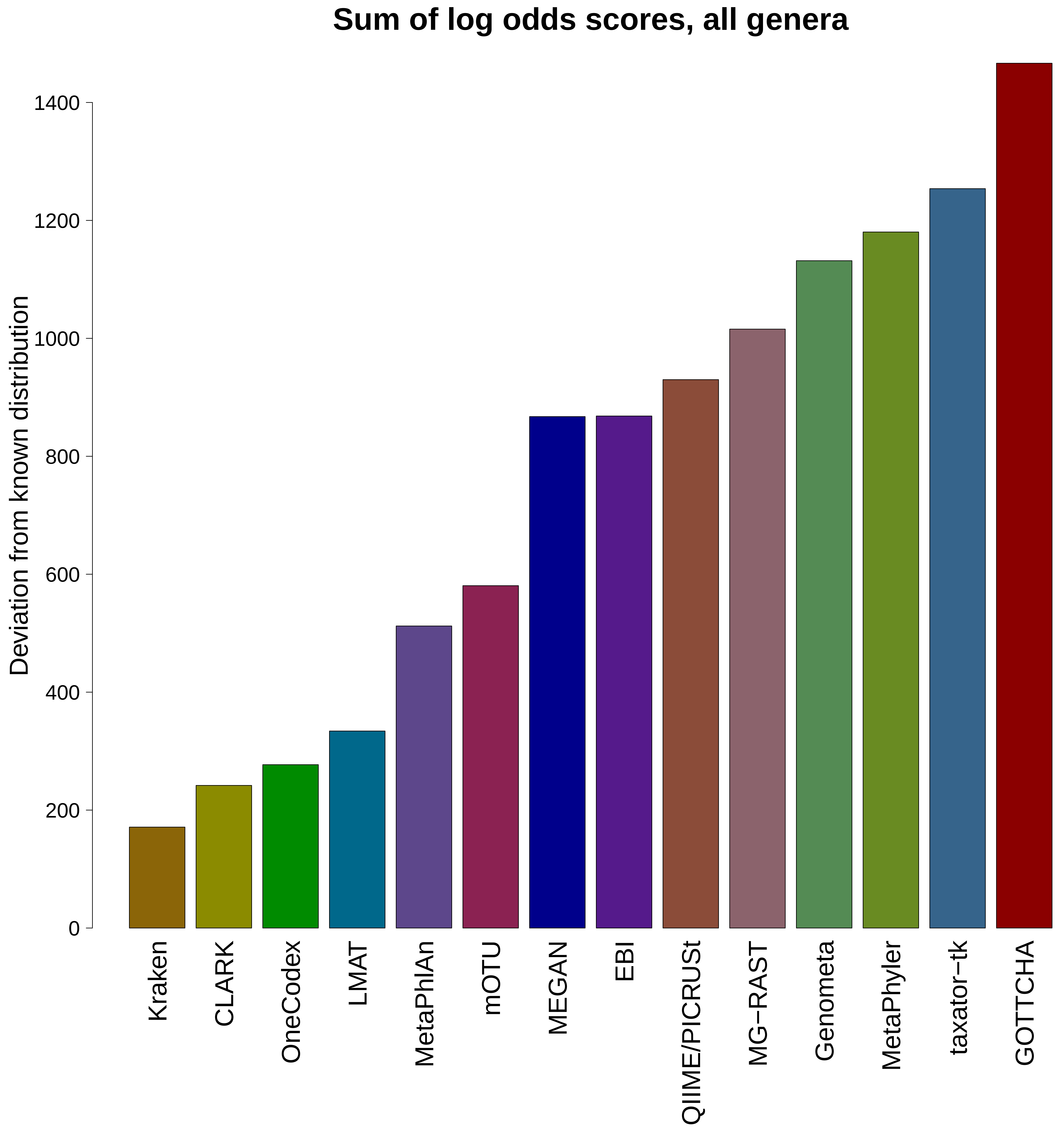
Sum of log odds scores, all genera
Deviation from known distribution
0
200
400
600
800
1000
1200
1400
Kraken
CLARK
OneCodex
LMAT
MetaPhlAn
mOTU
MEGAN
EBI
QIIME/PICRUSt
MG−RAST
Genometa
MetaPhyler
taxator−tk
GOTTCHA

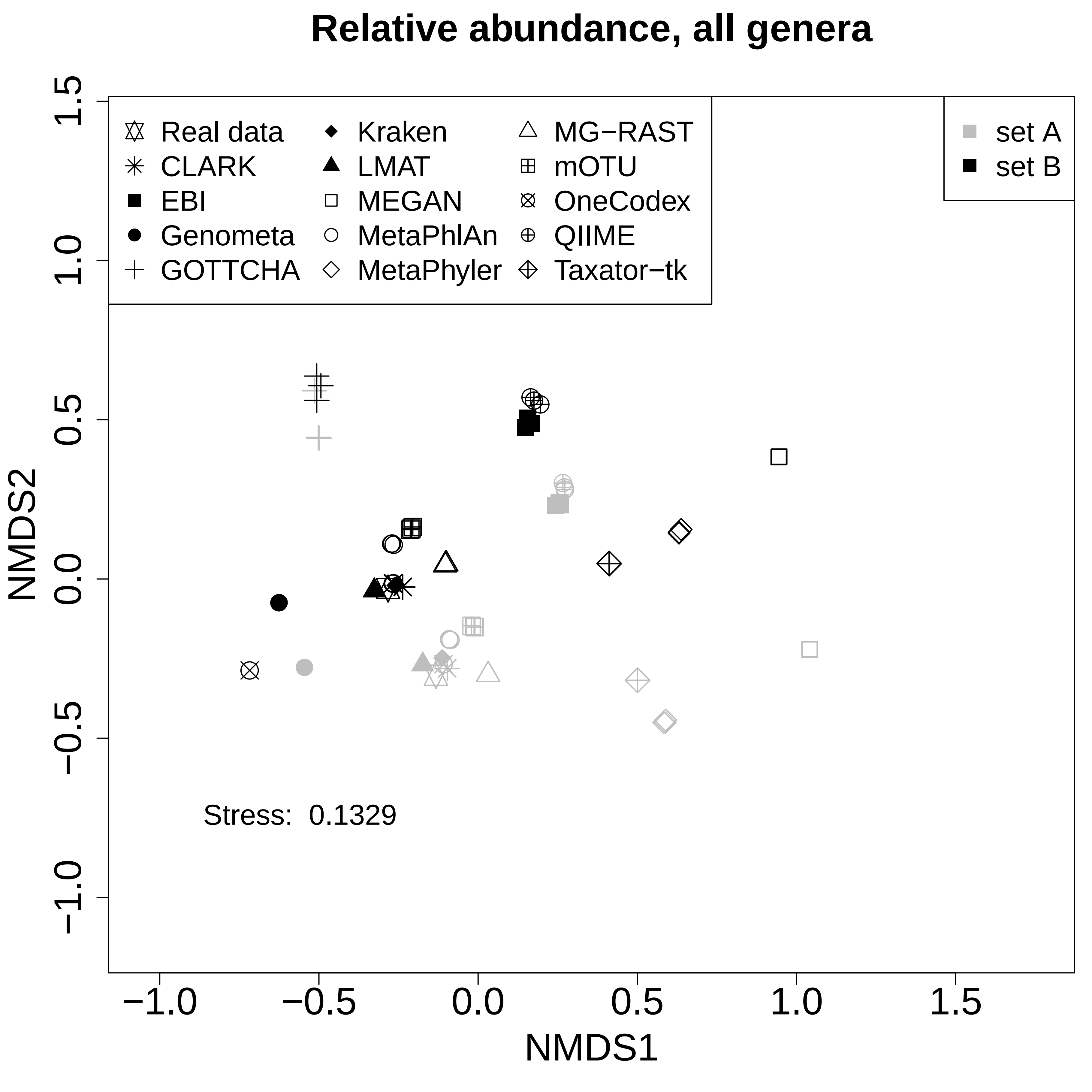

Relative abundance, all genera
Real data
CLARK
EBI
Genometa
GOTTCHA
Kraken
LMAT
MEGAN
MetaPhlAn
MetaPhyler
MG−RAST
mOTU
OneCodex
QIIME
Taxator−tk
set A
set B
Stress: 0.1329
NMDS1
NMDS2
−1.0
−0.5
0.0
0.5
1.0
1.5
